# Association between Obesity and Carotid Intima-Media Thickness in Korean Office Workers: The Mediating Effect of Physical Activity

**DOI:** 10.1155/2018/4285038

**Published:** 2018-08-01

**Authors:** Youngyun Jin, Donghyun Kim, Jinkyung Cho, Inhwan Lee, Kyuhwan Choi, Hyunsik Kang

**Affiliations:** College of Sport Science, Sungkyunkwan University, Suwon, Republic of Korea

## Abstract

**Background:**

Obesity and physical inactivity are associated with higher prevalence of cardiovascular disease (CVD).

**Objective:**

This study investigated the association between obesity and carotid intima-media thickness (CIMT) stratified by physical activity (PA) in Korean office workers.

**Methods:**

Data obtained from 914 office workers aged 21-60 years (347 women) were used. Resting blood pressures, body mass index (BMI), and waist-to-height ratio (WHtR) were measured. PA was assessed using the international physical activity questionnaire. CIMT was assessed with a carotid artery ultrasonography. Logistic regression analysis was used to estimate the odds ratio (OR) and 95% confidence interval (CI) of obesity stratified by weekly PA for an abnormally increased CIMT.

**Results:**

Logistic regression analyses showed that those who were overall obese (OR=2.50, 95% CI=1.60-3.91, P<0.001) or central obese (OR=2.08, 95% CI=1.29-3.40, P=0.003) had significantly higher estimated risks of having an abnormally increased CIMT even after adjustments for age, sex, smoking, alcohol consumption, resting blood pressures, and history of hypertension, diabetes, and hyperlipidemia, as compared with those who were not overall or central obese (OR=1). A multivariate linear regression suggested that age (P<0.001), sex (P=0.002), hypertension (P=0.014), smoking (P=0.054), BMI (P<0.001), and physical activity (P=0.011) were important determinants of abnormally elevated CIMT in this study population.

**Conclusion:**

The current findings suggest that the risk of obesity for an abnormally increased CIMT is significantly modulated by demographics as well as lifestyle-related risk factors including smoking and physical inactivity in Korean office workers.

## 1. Introduction

The Korean lifestyle is characterized as largely sedentary, with many people failing to engage in sufficient moderate or vigorous physical activity (PA) [1]. In particular, office workers in Korea spend most of their work hours in sedentary behaviors in conjunction with an unhealthy lifestyle featuring of heavy drinking and smoking and job-related stresses [2, 3]. Consequently, they are vulnerable to the development of metabolic and cardiovascular disorders, implying an urgent need for more attention and improvement.

Cardiovascular disease (CVD) is a leading cause of death in the general population. Carotid intima-media thickness (CIMT) holds prognostic information for future CVD [4] and is associated with the extent of atherosclerotic lesions [5]. An abnormally increased CIMT correlates with higher prevalence and incidence of CVD [6, 7]. On the other hand, a cholesterol-lowering therapy such as administration of statins may stabilize the carotid atherosclerosis [8] and/or regresses its progression [9].

Obesity is a major modifiable risk factor for CVD [10]. Body mass index (BMI) indicative of overall obesity is positively associated with an abnormally increased CIMT [11]. In addition, waist-to-height ratio (WHtR) indicative of body fat distribution is also closely associated with an abnormally increased CIMT as well as impaired arterial distensibility and compliance [12]. Along with obesity, low PA and sedentary behaviors are associated with higher CVD risk [13]. Thus, the world health organization (WHO) recognizes physical inactivity as the fourth leading risk factor for death globally, with an estimated 3.2 million deaths attributed to insufficient PA every year [10]. On the other hand, it is well known that regular PA has the potential to benefit cardiovascular health including atherosclerosis [14] as well as cardiovascular function, body composition, and metabolic conditions [15]

In Korea, office workers are known to be among the unhealthiest group of people due to their sedentarism characterized by prolonged sitting and lack of physical activity in conjunction with overweight and obesity [16]. However, there is a lack of research on office workers with respect to metabolic and cardiovascular health. Therefore, this study aimed to investigate the relationship between overall and central obesity and increased CIMT stratified by weekly PA levels in a sample of Korean office workers.

## 2. Methods

### 2.1. Subjects

In a cross-sectional design, a total of 982 adults (aged 21-60 years) voluntarily participated in this study, which was conducted as part of the annual health screening at local health centers between March 2015 and October 2016. The exclusion criteria included age of either younger than 21 years or older than 60 years, diagnosed CVD, and history of CVD or stroke. CVD was defined as present if the participant had a history of coronary artery disease, myocardial infraction, atrial fibrillation and other arrhythmias, and heart valve disease.

Data was collected during two separate visits. At the first visit, 589 men (8 missing) and 376 women (9 missing) completed demographic assessments, and medical history of hypertension, diabetes mellitus, and hyperlipidemia were obtained from a standardized self-administered questionnaire. The second visit involved an overnight fast after which laboratory tests were performed to evaluate anthropometrics, including height, weight, and waist circumference (WC), cardiovascular risk factors, and CIMT. During the second visit, 22 men and 29 women did not show up or refused to complete assessment of anthropometrics and risk factors or CIMT measurement. Consequently, 914 (567 men/347 women) completed all the measurements and were included in the final data analysis. Informed consent was obtained from all subjects prior to study participation. The Sungkyunkwan University Institutional Review Board, in accordance with the World Medical Association Declaration of Helsinki, approved the study protocol (SKKU 2015-09-001-005).

### 2.2. Assessment of Lifestyle Risk Factors

Lifestyle factors measured in this study included alcohol consumption (frequency per week) and smoking status. Smoking was defined as smoking more than 1 cigarette per day for at least 1 year, and participants were categorized as never smokers, ever smokers (ceased smoking for at least 6 months), and current smokers. Resting blood pressure (BP) was measured with an automatic BP instrument (Jawon Medical Co., Kyungsan, Republic of Korea) with subjects in seated position, with the arm at heart level and resting on the armrest of a chair.

### 2.3. Measurement of PA, Anthropometrics, and Obesity

The development of the International Physical Activity Questionnaire (IPAQ) commenced in Geneva in 1998 and was followed by extensive reliability and validity testing undertaken across countries [17]. The IPAQ is available in different languages at www.ipaq.ki.se (https://sites.google.com/site/theipaq/). In this study, a short Korean version of the IPAQ of which reliability and validity was tested and reported previously [18] was used. In brief, participants were asked about frequency (times/week) and duration (minutes) of PA lasting for at least 10 minutes according to intensity (light, moderate, and vigorous). Total PA per week was estimated by summing the minutes spent in moderate PA per week and twice the minutes spent in vigorous PA per week. The participants were classified as one of the two subgroups based on the WHO recommended minimum of weekly PA: (1) physically inactive (0-149 minutes of moderate PA per week or 0-74 minutes of vigorous PA per week or less than an equivalent combination of moderate-to-vigorous PA) or (2) physically active (≥150 minutes of moderate PA per week or ≥75 minutes of vigorous PA per week or an equivalent combination of moderate-to-vigorous PA).

Height and weight were measured with the subjects wearing light clothing and on shoes using an automate assessment equipment (DS-102, Jenix Co., Seoul, Republic of Korea) to the nearest decimal point. WC in cm was measured at midpoint between the bottom of rib cage and the top of the iliac crest using nonelastic tape. BMI (kg/m^2^) was calculated as body weight in kilograms divided by the square of height in meters. Waist-to-height ratio (WHtR) was calculated as the WC in cm divided by the height in cm. For data analyses, the subjects were then classified as either normal or obesity based on the BMI cutoff point of ≥25 kg/ m^2^ [19, 20], and they were also classified as either not central obese or central obese based on the WHtR cutoff point of ≥0.5 [21].

### 2.4. Measurement of CIMT

Subclinical carotid atherosclerosis was assessed using carotid artery ultrasonography equipped with a 7.5-MHz linear array imaging probe (Aloka ProSound Alpha 6, Hitachi Aloka Medical America, Wallingford, CT, USA). All measurements were performed by the same radiologist blinded to the subject's clinical status, with the subject in supine position, the head turned away from the side of interest, and the neck extended slightly. The measurement was performed in the longitudinal plan on both the right and left common carotid arteries, about 2-3cm proximal to the carotid bifurcation [20]. CIMT of the far wall was evaluated manually as the distance between the lumen-intima interface and the media-adventitia interface. Measurements were obtained manually from five contiguous sites at 12-mm intervals bilaterally, and the mean of measurements was used for statistical analysis. An abnormally increased CIMT was defined as a CIMT of the upper 25 percentile (≥0.71mm) in the entire study population

### 2.5. Statistical Analyses

All statistical analyses were performed using SPSS-PC version 20 (SPSS Inc., Chicago, IL, USA). Descriptive statistics of the demographic parameters were expressed as mean and standard deviation. Continuous variables are reported as means ± standard deviations, and categorical variables are presented as percentages. One-way analysis of variance followed by a post hoc test (LSD), if necessary, was used to compare measured parameters between BMI and WHtR-based subgroups stratified by weekly PA. Multivariable logistic regression was used to estimate odd ratio (OR) and 95% confidence interval (CI) of overall and central obesity stratified by weekly PA for an abnormally increased CIMT. Lastly, a multivariate linear regression analysis was conducted to identify significant determinants of an individual variation in CIMT in the study population. Statistical significance was accepted as p≤0.05.

## 3. Results

Table 1 represents the descriptive statistics of study participants according to gender. Overall, men had higher values in mean age (P<0.001), BMI (P<0.001), WHtR (P<0.001), percent body fat (P<0.001), LBM (P<0.001), resting systolic and diastolic BPs (P<0.001 and P<0.001, respectively), alcohol consumption (P<0.001), smoking rate (P<0.001), but higher rates of hypertension (P<0.001) and diabetes (P=0.009) in conjunction with a higher CIMT (P<0.001) than women.

Table 2 represents the measured parameters of BMI-based subgroups stratified by weekly PA. Among physically active individuals, those with BMI of ≥25 kg/m^2^ had higher values in WHtR (P<0.001), percent body fat (P<0.001), LBM (P<0.001), systolic and diastolic BPs (P<0.001 and P<0.001, respectively), smoking rate (P<0.001), and prevalence of hypertension (P=0.013) in conjunction with a higher CIMT than those with BMI of <25 kg/m^2^, with no statistically significance differences in mean age, alcohol consumption, and prevalence of diabetes and hypertension between the two subgroups. Among physically inactive individuals, those with BMI of ≥25 kg/m^2^ had higher values in mean age (P<0.001), WHtR (P<0.001), percent body fat (P<0.001), LBM (P<0.001), systolic and diastolic BPs (P<0.001 and P<0.001), smoking rate (P<0.001), alcohol consumption (P=0.002), and prevalence of hypertension (P=0.013) in conjunction with a higher CIMT (P<0.001) than BMI of <25 kg/m^2^, with no statistically significant difference in prevalence of diabetes and hyperlipidemia between the two subgroups.

Table 3 represents the measured parameters of WHtR-based subgroups stratified by weekly PA. Among physically active individuals, those with WHtR of ≥0.5 had higher values in mean age (P<0.001), BMI (P<0.001), percent body fat (P<0.001), LBM (P<0.001), systolic and diastolic BPs (P<0.001 and P=0.001, respectively), and smoking rate (P=0.012) in conjunction with a higher CIMT (P=0.001) than those with WHtR of <0.5, with no statistically significant differences in alcohol consumption and prevalence of hypertension, diabetes, and hyperlipidemia between the two subgroups. Among physically inactive individuals, those with WHtR of ≥0.5 had higher values in mean age (P<0.001), BMI (P<0.001), percent body fat (P<0.001), systolic and diastolic BPs (P<0.001 and P<0.001, respectively), smoking rate (P<0.001), alcohol consumption (P=0.022), and prevalence of hypertension (P<0.001) and hyperlipidemia (P<0.001) in conjunction with a higher CIMT (P<0.001) than those with WHtR of <0.5.

Table 4 represents the estimated risk of BMI-based obesity for subclinical atherosclerosis stratified by weekly PA. Among physically active individuals, there was no significant difference in the risk of subclinical atherosclerosis between BMI-based normal weight and obese groups. Among physically inactive individuals, however, BMI-based obese group had a significantly higher risk (OR=2.15, 95% CI=1.65-3.80, P<0.001) of subclinical atherosclerosis, as compared to BMI-based normal weight group. The increased risk of the obese group for subclinical atherosclerosis remained significant (OR=2.50, 95% CI=1.60-3.91, p<0.001) even after adjustments for age, WHtR, smoking, alcohol consumption, and prevalence of hypertension, diabetes, and hyperlipidemia.

Table 5 represents the estimated risk of WHtR-based central obesity for subclinical atherosclerosis stratified by weekly PA. Among physically active individuals, there was no significant difference in the risk of subclinical atherosclerosis between WHtR-based subgroups. Among physically inactive individuals; however, WHtR-based central obese group had a significantly higher risk of subclinical atherosclerosis (OR=2.31, 95% CI=1.46-3.66, P<0.001), as compared to WHtR-based no central obese group. The increased risk of the central obese group for subclinical atherosclerosis remained significant (OR=2.08, 95% CI=1.29-3.40, P=0.003) even after adjustments for age, BMI, smoking, alcohol consumption, hypertension, diabetes, and hyperlipidemia.

Table 6 represents the outcomes of a multivariate linear regression model for predicting CIMT. As a result, age (P<0.001), sex (P=0.002), hypertension (P=0.014), smoking (P=0.054), BMI (P<0.001), and physical activity (P=0.011) were found to be significant determinants of CIMT in the study population.

## 4. Discussion

In this cross-sectional study, we investigated the relationships of overall and central obesity with an abnormally elevated CIMT stratified by weekly PA in Korean office workers. In general, obese individuals, whether overall or central obese, had higher CVD risk factors, including higher mean age, higher body fatness, higher smoking and alcohol consumption, and higher resting blood pressures, than their nonobese counterparts. In particular, we found that the significantly higher risk of being overall or central obese for an abnormally elevated CIMT as a surrogate of subclinical atherosclerosis was found among physically inactive individuals only but not among physically active individuals, suggesting that the association between obesity and subclinical atherosclerosis is mediated by a lower PA, at least in this study population. Multivariate linear regression analyses showed that age, sex, smoking, hypertension, BMI, and physical activity were important determinants for predicting abnormally increased CIMT.

The current findings of the study support and extend those of previous studies reporting the risk of obesity for increased CIMT. CIMT correlates with BMI and WC/WHtR in children and adolescents [22], in middle-aged adults [23], and in older adults [24]. In a sample of Korean older women, Park et al. [25] also showed overweight and obese women had significantly higher risks of subclinical atherosclerosis (OR=3.033, 95%CI=1.064-8.650 and OR=2.459, 95%CI= 0.998-6.059, respectively) after adjustments for age, height, systolic blood pressure, physical activity level, and total cholesterol level, as compared with optimal weight women. In addition, central obesity was found to be more closely associated with an abnormally increased CIMT in middle-aged and elderly Chinese [24]. In the current study, likewise, we also found the independent and additive roles of BMI and WHtR in determining the risk of subclinical atherosclerosis in Korean office workers. Together, those findings suggest that, in addition to total body fat, body fat distribution should be considered in determining the risk of obesity for an abnormally increased CIMT.

With respect to PA and its relation to the atherosclerosis risk, Umbreen et al. [26] showed that leisure-time physical activity was significantly associated with CIMT in a cross-sectional study of 110 Pakistani adults (mean age of 58.6 ± 6.7 years). Galetta et al. [27] investigated the effect of physical activity on heart rate variability (HRV) and carotid intima-media thickness (IMT) by comparing 32 elderly sedentary subjects and 32 age-matched endurance athletes. In that study, they found that the elderly athletes had significantly lower CIMT than their sedentary counterparts. Regular PA was also found to delay the progression of CIMT in patients with coronary heart disease [28], in patients with stage 1 hypertension [29], and in patients with type 2 diabetes [30]. In support of the findings from previous cross-sectional and longitudinal studies, intervention studies have provided positive results on the ability of physical activity including regular exercise to reverse subclinical atherosclerosis in children [31, 32] as well as in older adults [33, 34]. For example, Woo et al. [32] investigated the effects of diet and/or exercise intervention programs in a sample of overweight children aged 9-12 years, and they found that obesity-related vascular dysfunction was partially reversed with the diet program alone and even more substantially with diet plus exercise program. Likewise, Park, Kwon, and Park [34] showed that 24 weeks of aerobic and resistance training resulted in significantly decreased CIMT and increased carotid flow velocity wall shear ration in a sample of overweight and obese older women. Together, these findings suggest that PA, including regular exercise, plays an important role in delaying and/or preventing the progression of atherosclerotic lesions in young and older subjects as well as in patients with CVD and metabolic disorders.

In particular, based on our knowledge on review of literatures, we are the first to report that the risk of obesity for an abnormally increased CIMT is significantly mediated by increased regular weekly PA in this study population of Korean office workers. Several explanations can be given for the mediating effect of weekly PA on the association between obesity and subclinical atherosclerosis. First, regular PA induces the beneficial effects on overweight/obesity, blood lipids, insulin resistance and diabetes, and hypertension [35], contributing to vascular health and reduced CVD incidence. Second, high cardiorespiratory fitness (CRF) provides a protection from being exposing to CVD risk and atherosclerotic lesions. Recent PA is associated with and may reflect individuals' CRF levels [36]. Consequently, the mediating effect of increased regular PA may reflect the benefits of promotion of CRF, CVD risk factors, and subclinical atherosclerosis [37]. On the other hand, lower levels of CRF are associated with morphologic and biochemical conditions that may reflect a primary atherosclerotic development [37]. Third, adipocytes produce a number of inflammatory cytokines, which may lead to systemic inflammation [38]. In this aspect, CIMT may represent an inflammatory process preceding the atheromatous plaque formation and atherosclerosis [39], which can be explained by the combination of negative profile of obesity and its related metabolic complications. On the other hand, regular PA may increase anti-inflammatory cytokines such as adiponectin and improve endothelial function [40]. Lastly, the mediating effect of regular PA on the association between obesity and increased CIMT may be a consequence of the nonatherosclerotic enlargement of carotid artery as an adaptive response to PA [41], through a nitric oxide-arteriogenesis-related mechanism [42]. Together, we speculate that the beneficial effects of regular PA on obesity, insulin resistance and metabolic complications, CVD risk factors, and inflammatory cytokines would collectively contribute to the alleviated risk of obesity for an abnormally increased CIMT as an early indicator of atherosclerosis.

This study has some limitations. Firstly, several major CVD risk factors such as blood lipids, insulin, and proinflammatory cytokines may play as additional modulators in determining the association between PA, obesity, and increased CIMT, and they should be accounted for in a future study. Secondly, the participants of the current study were all white collar office workers from Seoul, the capital city of Korea, who may not properly represent the overall office workers in Korea. Therefore, the current findings of the study may have a limitation in generalizing to Korean office workers. Thirdly, the cross-sectional nature of the current study did not allow us to suggest the exact mechanism(s) underlying those benefits, especially with respect to subclinical atherosclerosis, and this should be investigated in a future study. Lastly, the method of measuring CIMT manually used in this study may be subjected to bias and suboptimal reproducibility.

## 5. Conclusion

In this study, we investigated the association between overall and central obesity and CIMT stratified by week PA levels in Korean office workers and found that either overall or central obesity is significantly associated with increased CIMT among physical inactive individuals but not among physically active individuals. Finally, the current findings of the study suggest that, along with demographics (i.e., age and male sex) and hypertension, smoking, obesity, and physical inactivity may collectively contribute to subclinical atherosclerosis, implying an importance of promoting a healthy lifestyle for the prevention of atherosclerosis in this study population.

## Figures and Tables

**Table 1:** Characteristics of study participants.

Parameters	Total (n=914)	Men (n=567)	Women (n=347)	P value
Age, years	43.5±9.2	44.7±9.0	41.5±9.1	<0.001
BMI, kg/m^2^	24.1±2.9	25.1±2.6	22.4±2.6	<0.001
WHtR	0.51±0.04	0.52±0.04	0.50±0.05	<0.001
Body fat, %	25.9±6.1	23.2±5.0	30.5±5.1	<0.001
LBM, kg	27.9±6.2	32.0±3.9	21.2±2.3	<0.001
SBP, mmHg	123.2±14.6	128.0±13.2	115.4±13.4	<0.001
DBP, mmHg	78.9±10.5	82.7±9.1	72.6±9.7	<0.001
Smoking status, n (%)				
Never smoker	743(81.3)	399(70.4)	344(99.1)	<0.001
Former smoker	11(1.2)	10(1.8)	1(0.3)	<0.001
Current smoker	160(17.5)	158(29.8)	2(0.6)	<0.001
Alcohol consumption, n (%)				
No drinking	536(58.6)	342(60.3)	194(55.9)	<0.001
≤1 time per week	246(26.9)	122(21.5)	124(35.7)	0.002
≥2 time per week	132(14.4)	103(18.2)	29(8.4)	0.012
Hypertension, n (%)	99(11.1)	83(15.0)	16(4.7)	<0.001
Diabetes, n (%)	20(2.2)	18(3.3)	2(0.6)	0.009
Hyperlipidemia, n (%)	68(7.4)	49(8.9)	19(5.6)	0.070
CIMT, mm	0.52±0.10	0.54±0.11	0.50±0.80	<0.001

BMI: body mass index, LBM: lean body mass, WC: waist circumference, WHtR: waist-to-height ratio, SBP: systolic blood pressure, DBP: diastolic blood pressure, Regular PA: regular physical activity, and CIMT: carotid intima-media thickness.

**Table 2:** Characteristics of study participants according to physical activity and body mass index (BMI).

	Physically Active			P value	Physically Inactive			P value
	Total (n=333)	BMI (<25) (n=198)	BMI (≥25) (n=135)		Total (n=581)	BMI (<25) (n=383)	BMI (≥25) (n=198)	
Age, years	43.9±9.2	43.6±9.3	44.4±9.1	0.454	43.3±9.2	42.3±9.2	45.2±8.9	<0.001
BMI, kg/m^2^	24.3±2.6	22.6±1.6	26.8±1.5	<0.001	23.9±3.1	22.2±1.8	27.2±2.1	<0.001
WHtR	0.51±0.04	0.49±0.03	0.54±0.03	<0.001	0.51±0.05	0.49±0.04	0.55±0.03	<0.001
Body fat, %	24.6±6.0	23.3±5.8	26.6±5.8	<0.001	26.7±6.1	25.8±5.9	28.4±6.2	<0.001
LBM, kg	29.3±5.8	27.1±5.2	32.5±5.2	<0.001	27.1±6.3	24.8±5.3	31.6±5.6	<0.001
SBP, mmHg	123.6±13.3	121.0±13.8	127.5±11.7	<0.001	123.0±15.3	118.1±13.1	132.4±15.0	<0.001
DBP, mmHg	79.5±10.2	77.9±10.7	82.0±8.3	<0.001	78.5±10.8	75.4±10.1	84.6±9.4	<0.001
Current smoking, n (%)								
Never smoker	263	169(85.4)	94(69.7)	<0.001	480(82.6)	346(90.3)	134(67.7)	<0.001
Former smoker	4	1(0.5)	3(2.2)	0.149	7(1.2)	2(0.5)	5(2.5)	0.138
Current smoker	66	28(14.1)	38(28.1)	0.008	94(16.2)	35(9.1)	59(29.8)	<0.001
Alcohol consumption								
No drinking	117(53.2)	104(52.5)	73(54.1)	0.584	359(61.8)	236(61.6)	123(62.1)	0.589
≤1 time per week	99(29.7)	66(33.3)	33(24.4)	0.002	147(25.3)	109(28.5)	38(19.2)	0.011
≥2 time per week	57(17.1)	28(14.1)	29(21.5)	0.012	75(12.9)	38(9.9)	37(18.7)	0.012
Hypertension, n (%)	29(8.8)	11(5.6)	18(13.4)	0.013	70(12.4)	26(7.0)	44(22.7)	<0.001
Diabetes, n (%)	7(2.1)	5(2.5)	2(1.5)	0.516	13(2.3)	6(1.6)	7(3.6)	0.135
Hyperlipidemia, n (%)	20(6.0)	10(5.1)	10(7.5)	0.371	48(8.5)	27(7.3)	21(10.8)	0.154
CIMT, mm	0.52±0.08	0.51±0.08	0.53±0.08	<0.001	0.53±0.11	0.51±0.10	0.57±0.12	<0.001

Physically Active: meeting the WHO recommended minimum amount of weekly physical activity. Physically Inactive: not meeting the WHO recommended minimum amount of weekly physical activity. LBM: lean body mass, SBP: systolic blood pressure, DBP: diastolic blood pressure, and CIMT: carotid intima-media thickness.

**Table 3:** Characteristics of study participants according to physical activity and waist-to-height ratio (WHtR).

	Physically Active			P value	Physically Inactive			P value
	Total (n=333)	WHtR (<0.5) (n=134)	WHtR (≥0.5) (n=199)		Total (n=581)	WHtR (<0.5) (n=225)	WHtR (≥0.5) (n=356)	
Age, years	43.9±9.2	41.5±9.9	45.6±8.4	<0.001	43.2±9.2	40.7±9.4	44.8±8.8	<0.001
BMI, kg/m^2^	24.3±2.6	22.4±2.0	25.6±2.2	<0.001	23.9±3.1	21.5±2.0	25.5±2.6	<0.001
WHtR	0.51±0.04	0.47±0.02	0.54±0.03	<0.001	0.51±0.05	0.47±0.02	0.54±0.03	<0.001
Body fat, %	24.7±6.0	22.2±5.8	26.3±5.6	<0.001	26.7±6.1	24.3±5.7	28.3±5.9	<0.001
LBM, kg	29.3±5.8	27.8±5.9	30.3±5.5	<0.001	27.1±6.3	24.5±5.5	28.7±6.2	<0.001
SBP, mmHg	123.6±13.4	120.1±13.8	126.0±12.6	<0.001	123.0±15.3	116.1±12.6	127.4±15.3	<0.001
DBP, mmHg	79.4±10.0	77.3±11.0	80.9±9.1	0.001	78.4±10.8	74.5±10.5	81.0±10.2	<0.001
Current smoking, n (%)								
Never smoker	261(78.4)	114(85.1)	147(73.9)	0.012	482	199(88.4)	283(79.5)	0.018
Former smoker	4(1.2)	1(0.7)	3(1.5)	0.384	7	3(1.4)	4(1.1)	0.584
Current smoker	68(20.4)	19(14.2)	49(24.6)	0.004	92	23(10.2)	69(19.4)	0.012
Alcohol consumption								
No drinking	173(52.6)	79(59.0)	94(48.2)	0.012	354(61.5)	150(66.7)	204(58.1)	0.013
≤1 time per week	99(30.1)	35(26.1)	64(32.8)	0.042	147(25.5)	56(24.9)	91(25.9)	0.358
≥2 time per week	57(17.3)	20(14.9)	37(19.0)	0.102	75(13.0)	19(8.4)	56(16.0)	0.012
Hypertension, n (%)	29(8.9)	8(6.0)	21(10.8)	0.133	69(12.3)	8(3.7)	61(17.8)	<0.001
Diabetes, n (%)	7(2.1)	4(3.0)	3(1.5)	0.370	12(2.1)	3(1.4)	9(2.6)	0.327
Hyperlipidemia, n (%)	20(6.1)	4(3.0)	16(8.2)	0.052	48(8.6)	7(3.2)	41(12.0)	<0.001
CIMT, mm	0.52±0.08	0.50±0.08	0.53±0.08	0.001	0.53±0.11	0.49±0.09	0.55±0.12	<0.001

Physically Active: meeting the WHO recommended minimum amount of weekly physical activity. Physically Inactive: not meeting the WHO recommended minimum amount of weekly physical activity. LBM: lean body mass, SBP: systolic blood pressure, DBP: diastolic blood pressure, and CIMT: carotid intima-media thickness.

**Table 4:** Odds ratio (OR) and 95% confidence interval (CI) according to body mass index (BMI).

		Model 1	P value	Model 2	P value	Model 3	P value
Physically Active							
BMI	<25kg/m^2^	1 (reference)		1 (reference)		1 (reference)	
	≥25kg/m^2^	1.38 (0.81-2.35)	0.240	1.37 (0.80-2.34)	0.260	1.36 (0.78-2.36)	0.274
Physically Inactive							
BMI	<25kg/m^2^	1 (reference)		1 (reference)		1 (reference)	
	≥25kg/m^2^	2.51(1.65-3.80)	<0.001	2.65 (1.71-4.10)	<0.001	2.50 (1.60-3.91)	<0.001

Physically Active: meeting the WHO recommended minimum amount of weekly physical activity. Physically Inactive: not meeting the WHO recommended minimum amount of weekly physical activity.

Model 1 adjusted for age and sex.

Model 2 adjusted for Model 1 plus WHtR, smoking, and alcohol consumption.

Model 3 adjusted for Model 2 plus prevalence of hypertension, diabetes, and hyperlipidemia.

**Table 5:** Odds ratio (OR) and 95% confidence interval (CI) according to waist-to-height ratio (WHtR).

		Model 1	P value	Model 2	P value	Model 3	P value
Physically Active							
WHtR	≤0.50	1 (reference)		1 (reference)		1 (reference)	0.343
	>0.50	1.32(0.75-2.33)	0.338	1.30(0.73-2.31)	0.370	1.33(0.74-2.37)	
Physically Inactive							
WHtR	≤0.50	1 (reference)		1 (reference)		1 (reference)	
	>0.50	2.31(1.46-3.66)	<0.001	2.26(1.40-3.65)	0.001	2.08(1.29-3.40)	0.003

Physically Active: meeting the WHO recommended minimum amount of weekly physical activity. Physically Inactive: not meeting the WHO recommended minimum amount of weekly physical activity.

Model 1 adjusted for age and sex.

Model 2 adjusted for Model 1 plus BMI, smoking, and alcohol consumption.

Model 3 adjusted for Model 2 plus prevalence of hypertension, diabetes, and hyperlipidemia.

**Table 6:** Multivariate linear regression for predicting carotid-intima media thickness.

Variables	*β*	95% CI	P value
Age	0.399	0.004 ~ 0.005	<0.001
Sex	-0.045	-0.024 ~ 0.005	0.002
BMI	0.233	0.004 ~ 0.012	<0.001
WHtR	-0.046	-0.340 ~ 0.130	0.380
Hypertension	0.084	0.008 ~ 0.047	0.014
Smoking	0.101	-0.043 ~ -0.011	0.054
Physical activity	-0.086	-0.014 ~ -0.002	0.011

BMI: body mass index, WHtR: waist-to-height ratio, and smoking: past and current smokers.

Regression model F-value=54.438, adjusted R^2^=0.274, and P-value=0.001.

## Data Availability

The data used to support the findings of this study are included within the article.
